# Dynamic changes in copper homeostasis and post-transcriptional regulation of *Atp7a* during myogenic differentiation†
†Electronic supplementary information (ESI) available. See DOI: 10.1039/c7mt00324b


**DOI:** 10.1039/c7mt00324b

**Published:** 2018-01-04

**Authors:** Katherine E. Vest, Amanda L. Paskavitz, Joseph B. Lee, Teresita Padilla-Benavides

**Affiliations:** a Department of Biology , Emory University , 1510 Clifton Road , Atlanta , GA 30322 , USA; b Department of Biochemistry and Molecular Pharmacology , University of Massachusetts Medical School , 394 Plantation St. , Worcester , MA 01605 , USA . Email: Teresita.Padilla@umassmed.edu

## Abstract

Copper (Cu) is an essential metal required for activity of a number of redox active enzymes that participate in critical cellular pathways such as metabolism and cell signaling. Because it is also a toxic metal, Cu must be tightly controlled by a series of transporters and chaperone proteins that regulate Cu homeostasis. The critical nature of Cu is highlighted by the fact that mutations in Cu homeostasis genes cause pathologic conditions such as Menkes and Wilson diseases. While Cu homeostasis in highly affected tissues like the liver and brain is well understood, no study has probed the role of Cu in development of skeletal muscle, another tissue that often shows pathology in these conditions. Here, we found an increase in whole cell Cu content during differentiation of cultured immortalized or primary myoblasts derived from mouse satellite cells. We demonstrate that Cu is required for both proliferation and differentiation of primary myoblasts. We also show that a key Cu homeostasis gene, *Atp7a*, undergoes dynamic changes in expression during myogenic differentiation. Alternative polyadenylation and stability of *Atp7a* mRNA fluctuates with differentiation stage of the myoblasts, indicating post-transcriptional regulation of *Atp7a* that depends on the differentiation state. This is the first report of a requirement for Cu during myogenic differentiation and provides the basis for understanding the network of Cu transport associated with myogenesis.

## Abstract 2

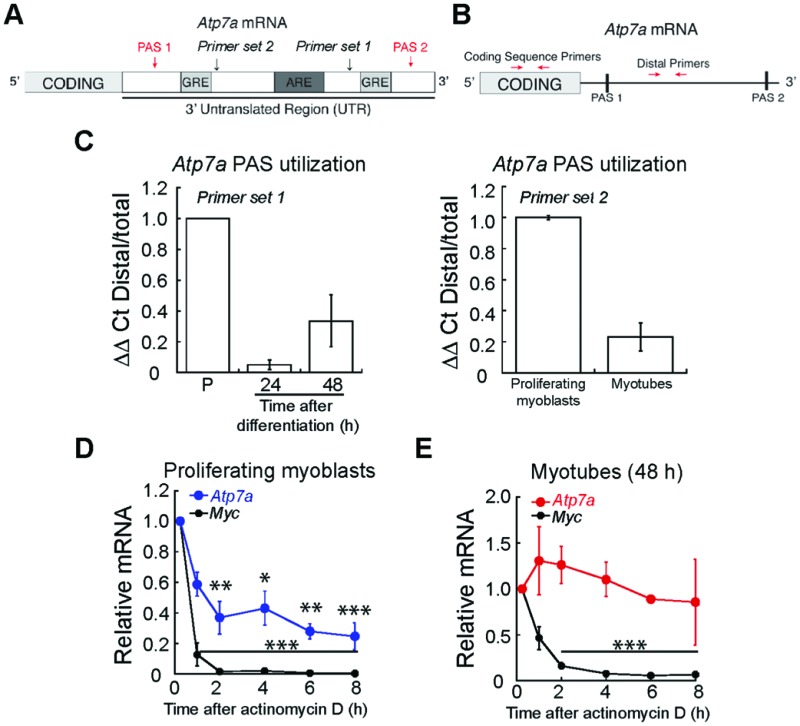
Copper (Cu) is an essential metal required for activity of a number of redox active enzymes that participate in critical cellular pathways such as metabolism and cell signaling.

##

Significance to metallomics

Copper is critical for development of mammalian cells. This micronutrient is particularly important for skeletal muscle differentiation and function, as this tissue intrinsically has a high demand for copper. Myogenesis encompasses a number of metabolic and morphological changes that have a strong link to copper biology, like energy production and redox homeostasis. This work demonstrates the copper requirement during myoblast proliferation and myogenic differentiation. Differential expression and localization of Cu transporters (CTR1, ATP7A, ATP7B) are orchestrated events that allow copper utilization and progression of myogenesis. This study set the basis to understand the role of copper in skeletal muscle development.

## Introduction

Transition metals are essential micronutrients that play important roles in basic cellular functions such as metabolism, gene expression, and stress response.^1^,^2^ Copper (Cu) is required as a cofactor for several enzymes including cytochrome *c* oxidase (COX), which is involved in mitochondrial ATP production, and superoxide dismutases (SOD1 and SOD3), which remove reactive oxygen species (ROS).^1^,^2^ Cu is also an important cofactor in enzymes that contribute to the establishment of cell tissue-specific functions.^3^ For example, in neurons, dopamine-β hydroxylase is necessary for norepinephrine synthesis and peptidyl–glycine–α-monooxygenase is required for the production of amidated neuropeptides.^4^,^5^ Lysyl oxidase (LOX), a secreted cuproenzyme, is required for stabilization of collagen fibrils and elasticity of elastin in the extracellular matrix.^6^

Though Cu is essential, it has the potential of participating in deleterious reactions such ROS production *via* Fenton chemistry and by interfering with the assembly of [Fe–S] cluster proteins.^7^–^9^ Therefore, cells utilize Cu-binding molecules and transporters to maintain low levels of free Cu and to ensure proper Cu distribution to different cellular destinations.^10^,^11^ Importantly, membrane barriers and transport proteins act as major regulators of Cu distribution. For example, the high affinity copper uptake protein 1 (CTR1) transports Cu into cells at the plasma membrane.^12^ The Cu-transporters ATP7A and ATP7B mediate the distribution of Cu to secreted cuproenzymes and Cu export.^13^–^20^

ATP7A and ATP7B are highly homologous transporters that belong to the P_1B_-family of Cu-ATPases. These are polytopic membrane proteins that mobilize cytosolic Cu using the energy derived from ATP hydrolysis.^18^,^21^,^22^ Both ATP7A and ATP7B have unique functions depending on cellular Cu status.^17^,^19^ The subcellular localization of Cu-ATPases in vesicles near the plasma membrane or in the *trans*-Golgi network determines whether they function in Cu export or in providing Cu to secreted cuproproteins.^17^ Both ATP7A and ATP7B play critical roles in regulating cellular and systemic Cu homeostasis, and mutations in the genes encoding these proteins cause Menkes disease and Wilson disease, respectively.^23^–^26^ Menkes disease patients experience systemic Cu deficiency, which leads to dramatic impairment of neurological development, blood vessel and connective tissue abnormalities, hypopigmentation, osteoporosis and hypotonia (weak muscle tone).^27^–^31^ Conversely, Wilson disease is characterized by Cu accumulation in the liver, brain, and eyes,^30^,^32^,^33^ which produces a wide variety of hepatic and neurological defects, osteoporosis, cardiomyopathies and neuromuscular phenotypes like ataxia (lack of coordination) or dystonia (repetitive movements).^34^,^35^ Additional allelic variants of *ATP7A* lead to other syndromes. For example, occipital horn syndrome is clinically and biochemically similar to Menkes disease, but with a less severe neurologic phenotype.^36^,^37^ Other pathogenic *ATP7A* alleles lead to an isolated distal motor neuropathy.^38^ Individuals affected by this neuropathy present with normal levels of *ATP7A* mRNA and protein and no signs of systemic Cu deficiency. However, a defect in ATP7A trafficking has been identified.^38^

The muscular symptoms observed in both Menkes and Wilson diseases are typically associated with motor neuron impairment. However, the contribution of muscular defects remains uncharacterized as the role of Cu in muscle development has rarely been studied. Due to the important functions of Cu in cell growth, survival, and metabolism, we hypothesized that Cu is critical for development of skeletal muscle. Using immortalized C2C12 myoblasts and primary myoblasts derived from mouse satellite cells, we detected an increase in whole cell Cu content during myogenic differentiation. We evaluated the cellular demands for Cu in murine myoblasts and found that Cu is necessary for both myoblast proliferation and myogenic differentiation. We demonstrate the dynamic expression of the Cu-ATPase ATP7A and show that *Atp7a* mRNA is subject to post-transcriptional regulation in myoblasts and myotubes. We also show that ATP7B is stably expressed in murine primary myoblasts and myotubes. This study demonstrates the importance of Cu in myogenic differentiation and provides a foundation for future studies to understand the roles for this trace metal in skeletal muscle development.

## Results

### Copper is necessary for myogenic differentiation

**Fig. 1:**
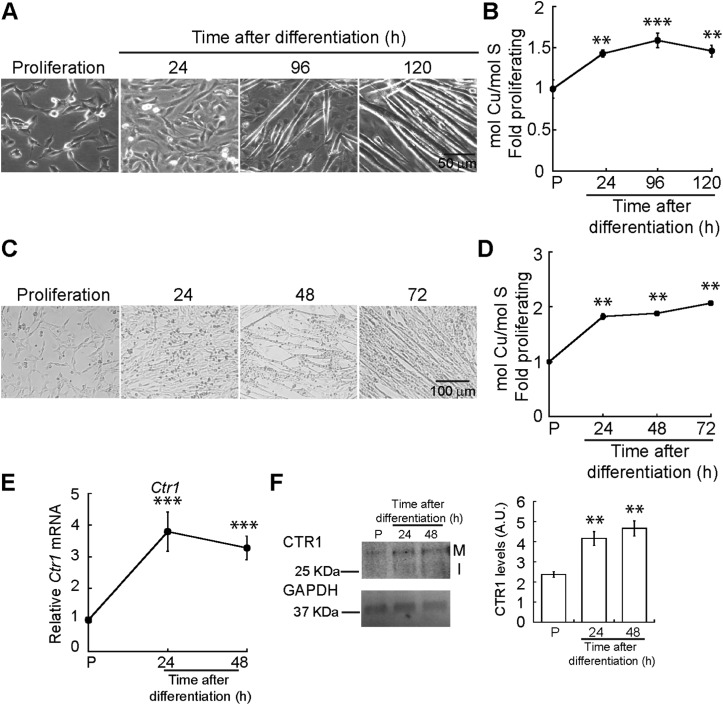
Cytosolic Cu increases during myogenic differentiation. (A) Representative light micrographs of proliferating and differentiating C2C12 myoblasts. (B) Whole cell Cu content of differentiating C2C12 myoblasts was determined by ICP-OES and reported as fold change relative to proliferating (P) cells. Data is represented as the average Cu concentration of three independent biological replicates ± standard error of the mean (SE). (C) Representative light microscopy images of proliferating and differentiating primary myoblasts derived from murine satellite cells. (D) Whole cell Cu content of differentiating primary myoblasts was determined by ICP-OES and reported as fold change relative to proliferating (P) cells. (E) Steady-state *Ctr1* mRNA levels in proliferating and differentiating primary myoblasts as determined by qRT-PCR. (F) CTR1 levels [primary translation product (I) and the mature glycosylated forms (M)] shown by representative Western blot and densitometric quantification of CTR1 bands in proliferating (P) and differentiating primary myoblasts of CTR1 in proliferating and differentiating primary myoblasts. GAPDH was used as loading control. Data are represented as the average Cu concentration of three independent biological replicates ±SE. **P < 0.01; ***P < 0.001.

Myogenesis encompasses a number of metabolic and morphological changes that are linked to cellular energy production and redox homeostasis, processes that require Cu.^1^,^2^ To explore the role of Cu in muscle development, we cultured C2C12 myoblasts in proliferating and differentiating conditions for 24, 96 and 120 h to obtain differentiating myocytes, nascent myotubes, and mature myotubes. Fig. 1A depicts representative light microscopy images of C2C12 myoblasts differentiated in culture. To determine whether differentiating myoblasts accumulate Cu, we measured whole cell Cu content using inductively coupled plasma-optical emissions spectroscopy (ICP-OES). A significant increase in cellular Cu was detected after 24 h of differentiation (Fig. 1B). Whole cell Cu increases as myogenesis progresses, and it peaks at 96 h post-differentiation. This increase in Cu levels was confirmed in primary myoblasts derived from murine satellite cells differentiated in culture to obtain nascent myotubes and mature myotubes (24 and 48 h; Fig. 1C). ICP-OES analysis confirmed a significant increase in intracellular Cu starting at 24 h after differentiation (Fig. 1D). CTR1 is the major Cu uptake transporter on the plasma membrane in eukaryotic cells. Steady-state mRNA (Fig. 1E) and protein (Fig. 1F) analyses showed an increase in *Ctr1* mRNA and protein upon initiation of myogenic differentiation, which may account for the intracellular increase of Cu. These results indicate dynamic Cu fluctuations associated with myogenic differentiation in C2C12 and primary myoblasts.

**Fig. 2:**
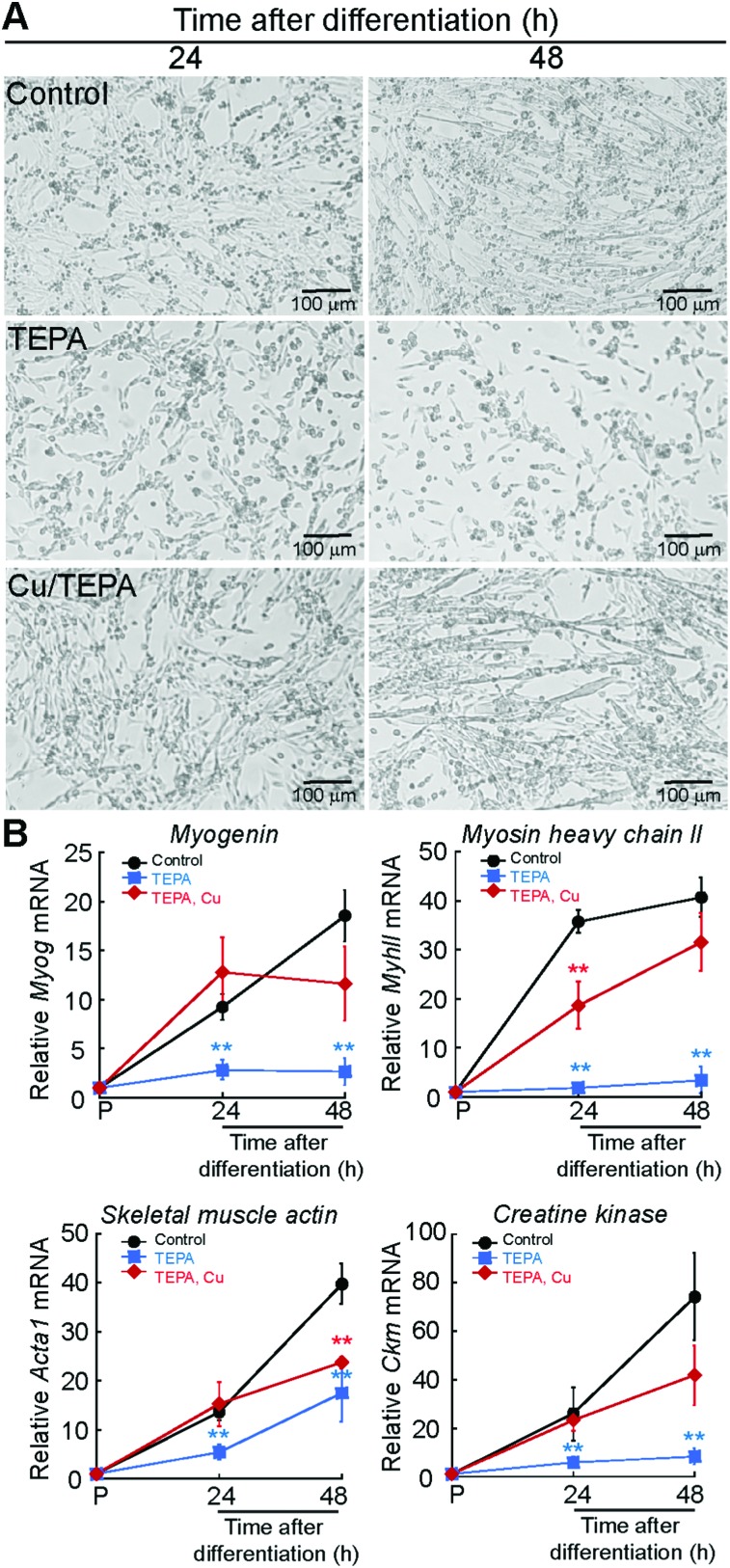
Cu chelation impairs myogenic differentiation. (A) Representative light micrographs of primary myoblasts derived from mouse satellite cells differentiated in media containing insulin (control), 30 μM TEPA, or 30 μM TEPA and 30 μM CuSO_4_ after 24 and 48 h. (B) Steady state mRNA levels of the myogenic markers *Myog*, *MyhII*, *Acta1* and *Ckm* in primary myoblasts differentiated as indicated in panel (A). Data represent the mean of at least four independent biological replicates ±SD. **P < 0.01.

We hypothesized that the increase in whole cell Cu levels in differentiating myoblasts is due to an inherent requirement for this metal during the myogenic program. To determine the Cu requirements for myogenesis, we differentiated primary myoblasts in the presence of the polyamine Cu chelator, tetraethylenepentamine (TEPA). The appropriate TEPA concentration was determined experimentally by differentiating primary myoblasts in the presence of increasing concentrations of TEPA (ESI† Fig. S1A). Representative light microscopy images showed that addition of 30 μM TEPA inhibits myotube formation in primary cells (Fig. 2A). To confirm impairment of myogenic differentiation with addition of TEPA, we quantified steady-state mRNA levels of the differentiation markers myogenin (*Myog*), myosin heavy chain II (*MyhII*), skeletal muscle actin (*Acta1*), and muscle specific creatine kinase (*Ckm*), using quantitative reverse transcriptase PCR (qRT-PCR) assay (Fig. 2B). We detected decreased steady state levels of all myogenic transcripts tested in cells differentiated in the presence of TEPA relative to untreated differentiated cells (Fig. 2B). Importantly, addition of exogenous Cu to TEPA-treated cells reverses differentiation defects associated with TEPA treatment (Fig. 2A and B). These data indicate that Cu is required for differentiation of primary myoblasts.

**Fig. 3:**
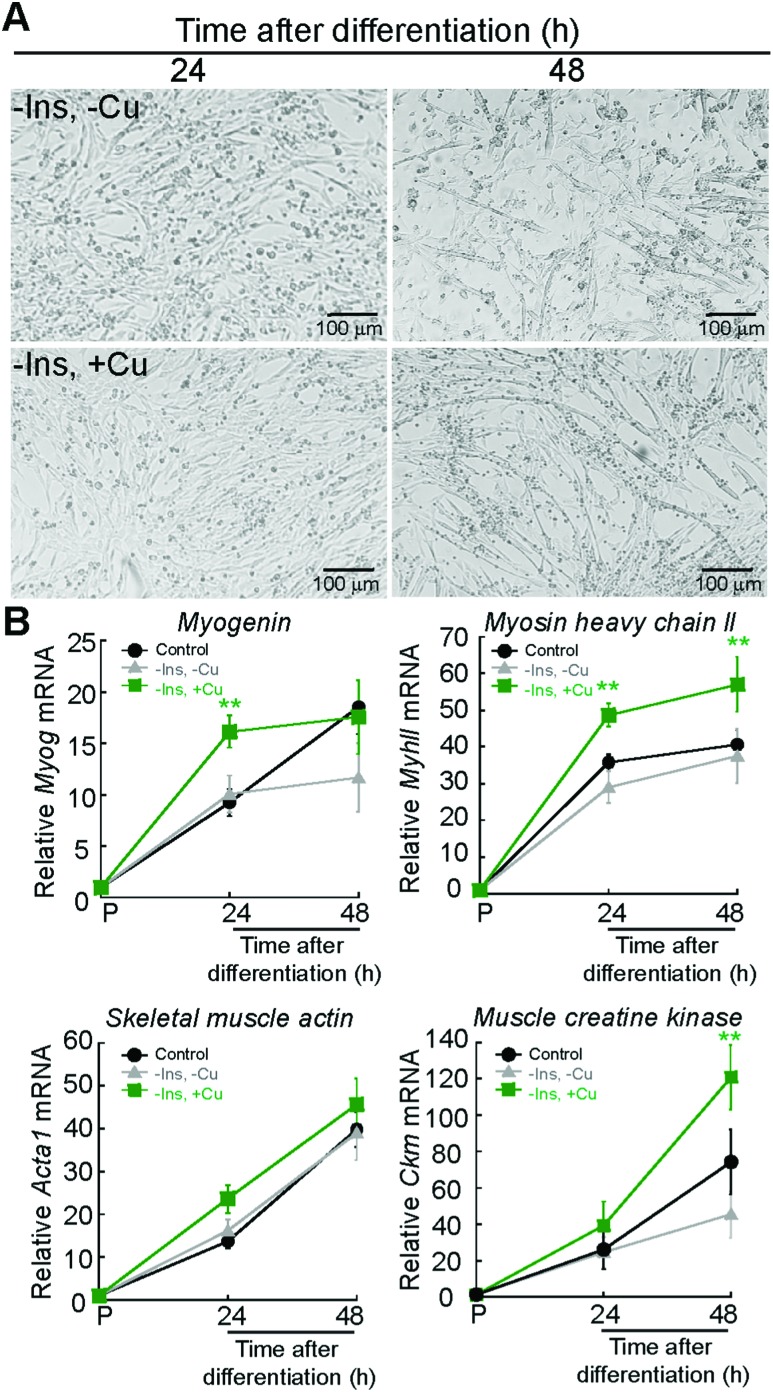
Exogenous Cu enhances myogenic differentiation. (A) Representative light micrographs of differentiating myoblasts the absence of insulin (top panel, –Ins, –Cu) and or in the absence of insulin but supplemented with 30 μM CuSO_4_ (bottom, –Ins, +Cu). (B) Steady state mRNA levels of the myogenic *Myog*, *MyhII*, *Acta1*, and *Ckm* in primary myoblasts cultured in the same conditions as indicate in panel (A). Control depicts the same control values used in Fig. 2. Data represent the mean of at least four independent biological replicates ±SD. **P < 0.01.

Protocols to induce myogenesis require depletion of growth factors from the media.^39^ This is achieved by starving the cells from growth factors using media supplemented with 2% horse serum. Addition of insulin to the differentiation media is necessary to induce myogenesis, as insulin activates signalling cascades such as the PI3K pathway.^39^,^40^ To test whether Cu can promote myogenesis, we eliminated insulin from the differentiation medium, a condition that partially prevents differentiation, as shown by light micrographs depicted in Fig. 3A. Addition of non-toxic concentrations of Cu (30 μM) to the insulin depleted medium rescues the differentiation defect (Fig. 3A). The effect of Cu on insulin signaling during myogenesis is currently under investigations and is beyond the scope of this paper. Non-toxic concentrations of Cu were determined experimentally by adding increasing concentrations of the metal to proliferating myoblasts (ESI† Fig. S1). Steady-state levels of myogenic mRNAs increase with Cu addition (Fig. 3B) when compared to myoblasts differentiated in the presence or absence of insulin (Fig. 3B). Together, these data suggest that Cu promotes myogenesis.

**Fig. 4:**
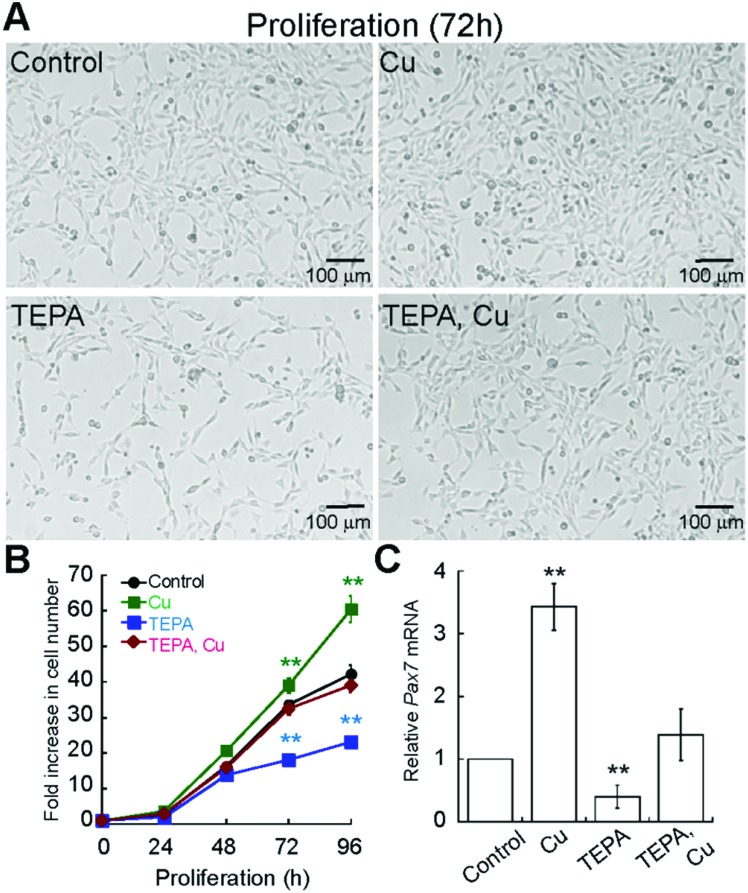
Cu chelation impairs primary myoblast growth. (A) Representative light micrographs of proliferating myoblasts grown in normal growth medium (control) or in the presence of Cu (100 μM CuSO_4_), TEPA (100 μM TEPA), and Cu/TEPA (100 μM TEPA and CuSO4). (B) Cell counting assay of primary myoblasts grown under the same culture conditions as in panel A. Data represent the average of three independent experiments ±SD. (C) Steady state *Pax7* mRNA levels in myoblasts cultured 48 h in the same growth conditions as in panel (A). All data represent the average of at least three independent biological experiments ± SD; **P < 0.01.

Considering that Cu enhances differentiation of primary myoblasts, we sought to determine whether Cu also affects myoblast proliferation. First, we determined the Cu and TEPA concentrations that were not toxic to the cells (ESI,† Fig. S1B and S2A). Importantly, the growth media used to maintain primary myoblasts contains 20% of FBS, which is rich in albumin, and other components that chelate Cu and other metals, so cells are able to grow in much higher Cu concentrations than those used for differentiation experiments. We grew cells in the presence of 100 μM TEPA or CuSO_4_ (Fig. 4A) and quantified proliferation by cell counting (Fig. 4B). We detected a significant decrease in cell numbers in the presence of TEPA and increased cell numbers in the presence of exogenous Cu (Fig. 4B). Addition of Cu to TEPA treated samples restores cell numbers to those of untreated cells (Fig. 4A and B). To assess a possible pathway by which Cu may influence primary myoblast proliferation, we investigated levels of *Pax7* mRNA. PAX7 is a transcriptional regulator expressed in proliferating muscle stem cells and it is necessary for myoblast proliferation.^41^–^46^ Myoblasts cultured for 48 h with CuSO_4_ have increased steady-state levels of *Pax7* mRNA while addition of TEPA leads to decreased *Pax7* mRNA levels (Fig. 4C). Addition of equimolar concentrations of CuSO_4_ to TEPA-treated cells restores *Pax7* mRNA levels, though not to levels detected in untreated cells (Fig. 4C). These data suggest that Cu enhances both proliferation and differentiation of primary myoblasts and that *Pax7* expression may be, in part, regulated by Cu.

Importantly, the proliferation and differentiation effects observed in Cu-cultured cells were specific to Cu treatment. Supplementation of normal growth media with ZnCl_2_ did not promote cell proliferation (ESI,† Fig. S2). Similarly, when differentiation was induced with increasing concentrations of ZnCl_2_ in the presence or absence of a chelator, we did not observe an increase in differentiation as detected for Cu (ESI,† Fig. S2). Other metal ions, were toxic (CoCl_2_ and NiCl_2_; not shown) or did not restore the differentiation defect (MnCl_2_ and MgCl_2_; not shown).

### Lineage specific expression and distribution of ATP7A and ATP7B in primary murine myoblasts

**Fig. 5:**
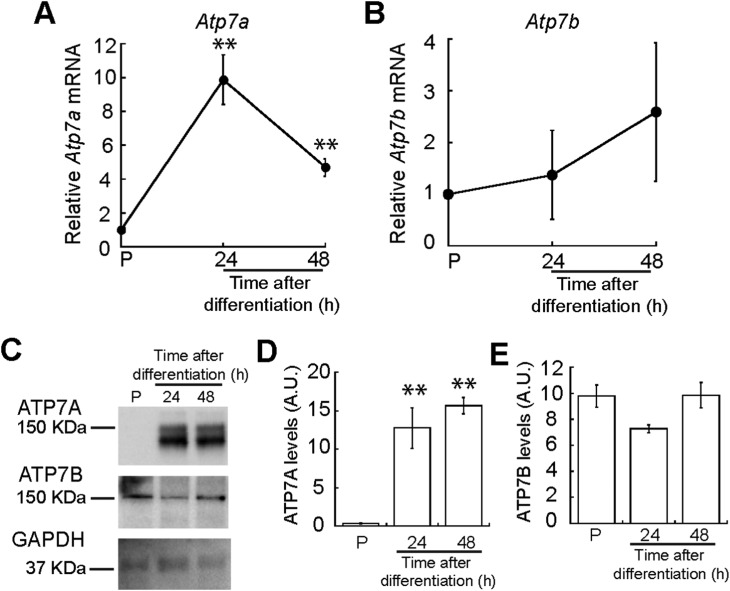
ATP7A and ATP7B expression in the myogenic lineage. (A) *Atp7a* expression is induced upon initiation of myogenic differentiation. Steady-state *Atp7a* mRNA levels in proliferating and differentiating primary myoblasts as determined by qRT-PCR. (B) *Atp7b* is constitutively expressed in proliferating and differentiating primary myoblasts. Steady state *Atp7b* mRNA levels in proliferating and differentiating primary myoblasts as determined by qRT-PCR. (C) ATP7A and ATP7B levels shown by representative Western blot and densitometric quantification of ATP7A (D) and ATP7B (E) bands in proliferating and differentiating primary myoblasts. GAPDH was used as loading control. All data represent the average of three independent biological experiments ± SD; **P < 0.01.

We hypothesized that myogenesis requires mobilization of Cu to the *trans*-Golgi network (TGN) as many secreted cuproenzymes are critical for differentiation of other progenitor cells.^47^,^48^ Therefore, we analysed expression of ATP7A, the Cu-ATPase that transports Cu into the TGN. We quantified *Atp7a* mRNA levels using qRT-PCR and detected increased steady-state levels of *Atp7a* mRNA that peak at 24 h of differentiation (Fig. 5A). To determine whether the increased mRNA levels correspond to increased protein levels, we performed immunoblots probing for ATP7A during myogenesis. Representative western blots and densitometric quantification revealed that ATP7A is absent from proliferating myoblasts, significantly increases 24 h after inducing differentiation, and remains elevated (Fig. 5C). This result indicates dynamic and differential expression of Atp7a during differentiation of primary myoblasts.

Although ATP7B is primarily a liver Cu-ATPase, microarray and RNA-seq experiments have detected *Atp7b* expression in other human tissues including skeletal muscle.^49^–^52^ We found that *Atp7b* mRNA and protein are stably expressed in proliferating and differentiating primary myoblasts (Fig. 5B–E), suggesting a constitutive need for this transporter in both myoblasts and myotubes.

**Fig. 6:**
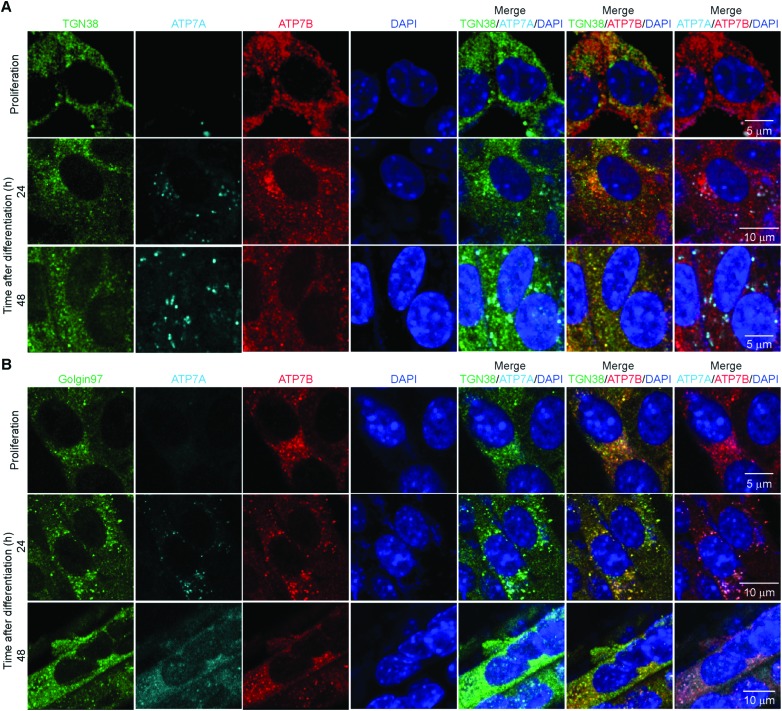
ATP7A and ATP7B co-localize differentially with the TGN markers TGN38 and Golgin-97. Representative confocal images of proliferating and differentiating myoblasts immunostained for: (A) TGN38 (green), ATP7A (cyan), ATP7b (red) and DAPI (blue). (B) Golgin-97 (green), ATP7A (cyan), ATP7b (red) and DAPI (blue). Images depicted are representative of three independent biological experiments.

Considering the differential patterns of expression of both Cu-ATPases, we asked whether these transporters also presented distinctive distribution in proliferating and differentiating primary myoblasts. Subcellular distribution of ATP7A determines its function. Localization of ATP7A to the TGN provides Cu for secreted cuproenzymes, while localization in vesicles close to the plasma membrane allows for Cu export when intracellular Cu concentrations are high.^3^,^53^,^54^ Confocal microscopy imaging of ATP7A in proliferating myoblasts revealed that this transporter is undetectable (Fig. 6) and is induced in differentiating cells. At 24h post-differentiation, ATP7A stains in a cytosolic spotted pattern near the nuclei which partially co-localizes with TGN38 and Golgin-97 in this area (Fig. 6).^47^,^55^ At later times, ATP7A appears to be distributed in cytosolic vesicles that co-localize primarily with Golgin-97. Co-localization of ATP7A with TGN38 and Golgin-97 suggests that this Cu-transporter is located in trafficking vesicles derived from Golgi. For instance, TGN38 is a resident integral membrane protein of the TGN that constitutively cycles between the TGN and the plasma membrane.^56^ TGN38 has been used as a model protein for the identification of post-Golgi trafficking motifs.^56^ Similarly, Golgin-97 is a resident protein of the Golgi apparatus proposed to be required for maintaining Golgi structure.^57^

Confocal microscopy analyses of ATP7B showed that this protein stains in punctate pattern in the cytoplasm that largely co-localizes with both, TGN38 and Golgin-97 in proliferating and differentiating myoblasts (Fig. 6). Interestingly, a vesicular pattern of ATP7B is also detected in the periphery of proliferating myoblasts, that does not co-localize with the neither of the TGN markers tested. These data suggest that ATP7B may be an important component for traffic and distribution and probably storage of Cu in proliferating and differentiating myoblasts. ATP7B has a vesicular non-polarized pattern in proliferating myoblasts that partially changes to a more perinuclear distribution in differentiated myoblasts. A similar cytosolic vesicular pattern for ATP7B has been observed in the intestine.^58^ The staining pattern of ATP7B in these cell types is different to the classic polarized staining in hepatocytes.^59^ In enterocytes, ATP7B vesicles have been proposed as Cu storage components which may subsequently be released by ATP7A.^58^ To test this hypothesis, we analysed whether ATP7A and ATP7B co-localize in differentiating myoblasts. Representative confocal imaging (Fig. 6) showed a partial co-localization of ATP7A and ATP7B in perinuclear vesicles. Negative controls lacking primary anti-ATP7A and anti-ATP7B but including the species specific fluorescent secondary antibodies show no immunostaining for either ATPase (ESI† Fig. S3A).

### Expression of *Atp7a* is regulated at a post-transcriptional level in primary murine myoblasts

**Fig. 7:**
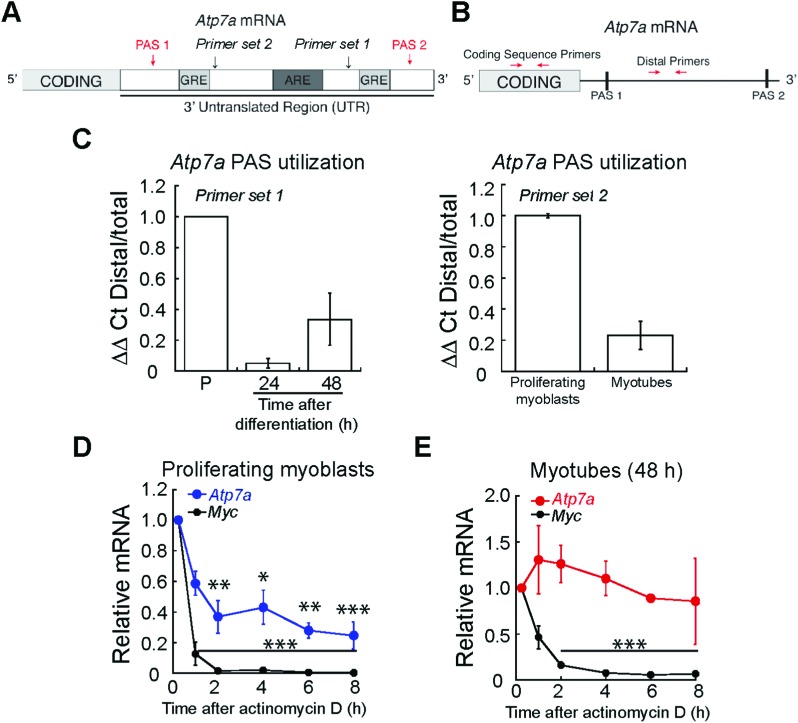
*Atp7a* mRNA is regulated at the post-transcriptional level during myogenic differentiation. (A) Schematic of *Atp7a* 3′ UTR showing putative PAS, GREs, and ARE. (B) Schematic of qRT-PCR strategy for quantifying PAS utilization in *Atp7a* mRNA. (C) Altered PAS utilization in *Atp7a* during myogenic differentiation as determined by qRT-PCR assay indicated in panel (B). (D) *Atp7a* mRNA in proliferating myoblasts is unstable as determined by actinomycin D treatment followed by qRT-PCR. Stability of *Myc* mRNA was used as a positive control. (E) *Atp7a* mRNA in differentiated myotubes is stable as determined by actinomycin D treatment followed by qRT-PCR. Stability of *Myc* mRNA was used as a positive control. All data represent the average of three independent biological experiments ± SE; *P < 0.05, **P < 0.01, ***P < 0.001.

While post-translational regulation of ATP7A has been well-described,^60^ few studies have probed post-transcriptional regulation of *Atp7a* mRNA. A major site of regulation is the 3′ untranslated region (3′ UTR), which contains multiple *cis*-acting elements.^61^ We studied the sequence of the *Atp7a* 3′ UTR and identified putative AU-rich elements (ARE) and GU-rich elements (GRE) between two polyadenylation signals (PAS) that regulate the site of polyadenylation and cleavage in the 3′ UTR (Fig. 7A). As shown in the schematic in Fig. 7B, we used qRT-PCR to assay PAS utilization in *Atp7a* by designing primers to the region between the two PAS sequences (Distal Primers, ESI† Table S1) and compared Distal PCR products to the total mRNA pool quantified by primers targeting the coding sequence (Coding Sequence Primers, ESI† Table S1). In differentiated myotubes, we detected a decrease in the steady-state levels of the distal PCR product relative to the total mRNA pool (Fig. 7C) when compared to proliferating myoblasts. This result suggests the presence of a shorter 3′ UTR and increased utilization of the more proximal PAS. To confirm that the 3′UTR of *Atp7a* is shortened during myogenic differentiation, we tested an additional primer set and detected decreased distal PCR product in myotubes relative to proliferating myoblasts (Fig. 7C). The region between the proximal and distal PAS contains *cis*-regulatory elements that act as binding sites for RNA binding proteins and micro RNAs that can affect mRNA turnover. Therefore, we assayed *Atp7a* stability in proliferating myoblasts *vs.* differentiated myotubes by inhibiting transcription with actinomycin D, and evaluated *Atp7a* mRNA levels by qRT-PCR.^62^ Steady-state levels of *Atp7a* in proliferating myoblasts significantly decreases by 50% after 2 h of actinomycin D treatment (Fig. 7D). In differentiated myotubes, *Atp7a* mRNA levels did not decrease at all time points tested (Fig. 7E). Assay for stability of *Myc*, an unstable mRNA, was used as a positive control (Fig. 7D and E). Taken together, these data indicate dynamic regulation of *Atp7a* mRNA stability during myogenic differentiation.

Because the levels of ATP7B do not change during myogenic differentiation, we did not expect to detect significant post-transcriptional regulation of *Atp7b* mRNA. Analysis of the *Atp7b* 3′ UTR revealed that it contains only one PAS (ESI† Fig. S4A). Actinomycin D treatment followed by qRT-PCR for *Atp7b* revealed no significant change this mRNA up to 8 h post actinomycin D treatment, in both myoblasts and myotubes (ESI† Fig. S4B and C). Assay for stability of *Myc*, an unstable mRNA, was used as a positive control (ESI† Fig. S4B and C). This result indicates that *Atp7b* mRNA is stable in both myoblasts and myotubes, and is likely not regulated at the level of RNA stability in primary murine muscle cells. The differential expression and localization of both Cu-exporters further supports a specific role and differential targeting of Cu during myogenesis, potentially associated to the secretory pathway, as shown for other differentiation models.^47^

## Discussion

We sought to determine how impaired myogenesis may contribute to pathology of Cu homeostasis diseases by studying Cu handling during myogenic differentiation in cultured cells. Using ICP-OES, we detected an increase in cellular Cu levels during differentiation of immortalized C2C12 and primary myoblasts derived from mouse satellite cells. Considering that expression of the high affinity Cu importer CTR1 is induced during myogenesis, it is plausible that the increase of Cu during myogenesis is dependent on the activity of CTR1. We found that Cu chelation with TEPA impaired differentiation while addition of exogenous Cu enhanced differentiation. In addition to the role of CTR1 in Cu import during myogenesis, we hypothesized that intracellular Cu is utilized, in part, for loading of secreted cuproenzymes in the TGN, a process largely driven by Cu-ATPases. Indeed, we detected dynamic expression of ATP7A that is regulated at the level of RNA stability during myogenic differentiation. We also detected a constitutive expression of ATP7B, which had not been shown previously in myoblasts. This is the first study showing the Cu requirement for myogenic differentiation and of post-transcriptional regulation of an important Cu homeostasis gene by alternative polyadenylation and RNA stability in myoblasts.

To understand the role for Cu in myogenesis, we analysed the Cu levels during differentiation of C2C12 and primary myoblasts. We found that Cu increases by ∼50% in early stages of differentiation corresponding to the time of formation of nascent myotubes. In both systems, Cu levels decreased slightly in mature myotubes. This plateau in Cu accumulation is likely because Cu is tightly regulated and homeostatic mechanisms are activated to prevent further accumulation beyond the 50% increase. We found that Cu influx is necessary for myogenic differentiation as Cu chelation with TEPA led to decreased steady-state levels of mRNAs encoding myogenic proteins. Addition of exogenous Cu enhanced differentiation and proliferation of primary myoblasts, suggesting that Cu may play multiple roles in myoblast function.

We show that, similar to neuronal differentiation,^47^ myogenic differentiation is associated with increased *Atp7a* mRNA and protein. ATP7A immunostaining shows a punctate perinuclear pattern consistent with partial localization to elements of the TGN, suggesting that Cu delivery to secreted cuproenzymes or potentially for storage, may be important for normal differentiation of myoblasts. A likely target for metallation by ATP7A is LOX, which crosslinks collagen and elastin and is thus critical for extracellular matrix deposition.^63^ LOX is important for differentiation of a number of cell types including chondrocytes,^64^ myofibroblasts,^65^ and osteoblasts.^66^ Interestingly, satellite cells also contribute to the formation and remodelling of the extracellular matrix, suggesting that LOX expression in satellite cells may be critical their function *in vivo.*^67^ Importantly, LOX is also involved in regulating muscle structure and function *via* feedback loop with TGF-β.^68^ Whether the dynamic expression of ATP7A and potential role of this Cu-ATPase to provide Cu for secreted cuproenzymes like LOX remains to be elucidated; however it is tempting to hypothesize that ATP7A is potentially a critical component of muscle development and regeneration.

Transcriptional and post-translational regulation of ATP7A has been well-described.^69^–^72^ However few studies have characterized regulation of the *Atp7a* mRNA, which could allow cells to fine tune ATP7A expression. Analysis of the *Atp7a* 3′ UTR revealed the presence of multiple putative polyadenylation and cleavage signals. We detected evidence of a shift in *Atp7a* PAS utilization during differentiation from a more distal PAS to a more proximal PAS. This result is consistent shortening of the 3′ UTR and a corresponding loss of *cis*-acting regulatory elements.^73^ Indeed, along with increased steady-state levels of *Atp7a* in differentiating myotubes, we detected increased stability of *Atp7a* mRNA in differentiated myotubes relative to proliferating myoblasts. The distal portion of the 3′ UTR, between proximal and distal PAS sites, contains putative AU-rich and GU-rich elements, which may serve as binding sites for proteins that mediate RNA degradation in proliferating myoblasts. The identity of these proteins remains unknown, but several RNA binding proteins such as argonaute-2 (AGO2), human antigen R (HuR), and cleavage and stimulation factor 2 (CSTF2) have been shown to interact with the human *ATP7A* 3′UTR in various cross-linking immunoprecipitation (CLIP) sequencing studies.^74^–^76^ AGO2 is involved in microRNA-mediated gene silencing^74^ while HuR has been shown to mediate either stabilization or destabilization of RNAs.^77^,^78^ CSTF2 is a component of the cleavage and polyadenylation machinery, which has been shown to regulate PAS utilization of several RNAs in C2C12 myoblasts including *Atp7a.*^79^ The functions of these cleavage and polyadenylation regulatory proteins in Cu homeostasis remain to be elucidated but alternative PAS utilization and 3′ end regulation may represent a new mechanism by which cells maintain Cu homeostasis.

The second mammalian Cu-transporting P-type ATPase gene, *Atp7b*, is mutated in the Cu overload associated with Wilson disease. Though ATP7B is most highly expressed in the liver, it is expressed in other tissues including neurons, kidney, and skeletal muscle.^24^,^25^,^80^–^82^ Here, we demonstrate for the first time that ATP7B is expressed in satellite cell derived myoblasts and in myotubes differentiated in culture. Confocal microscopy analyses showed that ATP7B largely co-localizes with TGN38, suggesting also a potential role for this transported in providing Cu to secreted cuproenzymes. A novel role for ATP7B for Cu storage has been recently described in enterocytes.^58^ Considering ATP7B constitutive expression and the vesicular non-polarized pattern observed here, it is plausible that this Cu-ATPase also contributes to Cu storage in the myogenic lineage. Though Wilson disease primarily affects the liver and brain, there is potential neuromuscular involvement. For example, some affected individuals experience muscle spasm and weakness.^83^ Interestingly, many Wilson disease patients experience swallowing difficulty, which is dependent on skeletal muscle function, before developing other neurologic symptoms.^84^ The stable expression of ATP7B during myogenic differentiation, suggest that there is a basal requirement for ATp7B in both proliferating myoblasts and differentiated muscle cells, but future experiments are required to identify the key functions of ATP7B in muscle cells.

## Conclusions

This work demonstrates that copper is a critical component of muscle cell differentiation and that a key Cu homeostasis gene, *Atp7a*, is regulated by alternative polyadenylation and RNA stability. Considering that *Pax7* expressing muscle progenitor cells play a critical role in embryonic muscle development, early postnatal muscle growth and may contribute to adult muscle maintenance,^41^,^45^,^46^,^85^–^91^ loss of copper homeostasis may impact both muscle development and adult muscle function. Thus, pathology of Cu homeostasis diseases may have a previously uncharacterized satellite cell component. For example, impaired satellite cells due to loss of ATP7A function in Menkes disease may impair muscle development leading to hypotonia. A similar model of hypotonia in Fragile X Syndrome was recently hypothesized when the fragile X mental retardation protein was shown to be critical for translational regulation in satellite cells.^92^ This study demonstrates an important role for Cu homeostasis in muscle progenitor cell function and provides the basis for future studies linking muscle progenitors and Cu homeostasis diseases.

## Experimental procedures

### Primary cell culture

Mice were housed in the animal care facility at the University of Massachusetts Medical School (Worcester, MA, USA) and at the Emory University School of Medicine (Atlanta, GA, USA) in accordance with the Institutional Animal Care and Use Committee guidelines. Mouse satellite cells were purified from whole leg muscle from 3- to 6-week-old male and female wild type C57Bl/6. Differential plating following Purcol sedimentation was performed as previously described.^93^ Briefly, muscle tissue was excised, rinsed with Hank's Balanced Salt Solution (HBSS; Life Technologies), sliced into small pieces and incubated with 0.1% Pronase in HBSS at 37 °C for 60 min. The cell suspension was filtered with a 100 μm cell sieve and re-suspended in 3 ml of normal growth media containing 1 : 1 mix of DMEM and F-10, 20% fetal bovine serum (FBS) and 25 ng ml^–1^ of recombinant basic FGF (Millipore). Cells were filtered using a 40 μm cell sieve and centrifuged at 1000 × *g* for 1 min at room temperature. The cells were placed in the top of the Percoll gradient (35 and 70%) and centrifuged 20 min at 1850 × *g* at room temperature. The myoblasts were obtained from the lower interface of the 70% Percoll fraction. Cells were washed with HBSS, centrifuged 5 min at 1000 × *g*, and re-suspended and plated in growth media. Isolated myoblasts were grown on plates coated with 0.02% collagen (Advanced BioMatrix).

Primary myoblasts were plated at 4 × 10^4^ cells per cm^2^ for further experimentation. Cells were maintained in growth media (GM: 1 : 1 mixture of DMEM : Ham's F10, 20% FBS, 5 ng ml^–1^ bFGF, 100 U ml^–1^ penicillin G, 100 mg ml^–1^ streptomycin) in a humidified 5% CO_2_ incubator at 37 °C on collagen-coated dishes. The differentiation media (DM) contained: DMEM, 2% Horse Serum (HS), 100 U ml^–1^ penicillin and 100 mg ml^–1^ streptomycin, and 1% Insulin–Transferrin–Selenium-A supplement (Invitrogen) was added as needed (indicated in figure legends). Cells were grown and differentiated with increasing concentrations CuSO_4_ (10–500 μM) and/or the Cu chelator tetraethylenepentamine (TEPA, 10–50 μM) as indicated in the figures. For all experiments, at least three independent isolates were analysed.

C2C12 cells were purchased from American Type Culture Collection (CRL-1772). Differentiation of C2C12 cells was initiated by plating 4 × 10^4^ in DMEM media containing 10% FBS. After 48 h, cells reached confluence; the media was changed to differentiation media and were allowed to differentiate for up to 7 days. Different time points were obtained as indicated in the figures. For all experiments, at least three independent isolates were analysed.

### Whole-cell metal content

Proliferating and differentiated primary myoblasts derived from mouse satellite cells and the established cell line C2C12 were washed three times in 5 ml of buffer containing 50 mM HEPES (pH 7.5) and 500 mM NaCl (all glassware was previously rinsed with deionized H_2_O containing 5% HNO_3_, trace metal grade). The samples were resuspended in concentrated HNO_3_ (trace metal grade) and analysed by inductively coupled plasma-optical emissions spectroscopy (ICP-OES) as previously described.^94^ In all cases, metal concentrations were normalized to the concentration of sulphur, which is correlated with total protein concentration and can be used in place of quantification per protein.^95^ Values are reported as fold change relative to proliferating cells.

### Antibodies

Primary antibodies used were rabbit anti-CTR1 (FL-190, sc-66847, Santa Cruz) mouse anti-TGN38 (MA3-063, Thermo Fisher Scientific), mouse anti-golgin-97 (A21270, Life Technologies), rabbit anti-ATP7A (H-180 sc-32900, Santa Cruz), goat anti-ATP7B (K13, sc-32446, Santa Cruz), Mouse anti-GAPDH (G9295, Sigma). Secondary antibodies used were donkey anti-mouse Alexa-488, donkey anti-rabbit Alexa-633, and donkey anti-goat Alexa 594 and HRP-conjugated anti-mouse, anti-rabbit (Thermo Fisher Scientific), and anti-goat secondary antibodies (Pierce).

### Western blot analysis

Proliferating primary myoblasts were washed with PBS and solubilized with RIPA buffer (10 mM piperazine-*N*,*N*-bis(2-ethanesulfonic acid), pH 7.4, 150 mM NaCl, 2 mM ethylenediamine-tetraacetic acid (EDTA), 1% Triton X-100, 0.5% sodium deoxycholate, and 10% glycerol) containing complete protease inhibitor cocktail. Protein content was quantified by Bradford.^96^ Thirty micrograms of each sample were prepared for SDS-PAGE by boiling in Laemmli buffer.^97^ The resolved proteins were electrotransferred to PVDF membranes (Millipore). The proteins of interest were detected with the specific polyclonal or monoclonal antibodies indicated, followed by species – appropriate peroxidase – conjugated secondary antibodies and chemiluminescent detection (ECL PLUS; GE Healthcare).

### Primary myoblast immunofluorescence and confocal analysis

Cells for immunofluorescence were grown on glass bottom Cellview Advanced TC culture dishes (Grenier Bio One). Samples were obtained for proliferation and different time points after induction of differentiation in the presence and absence of 30 μM CuSO_4_ and 30 μM TEPA. Cells were fixed in 10% formalin, blocked in 5% horse serum, 0.2% Triton X-100 in PBS, then incubated overnight with primary antibodies at 4 °C diluted 1 : 100 in blocking buffer. Cells were washed three times with 0.2% Triton X-100 in PBS and sequentially incubated for 2 h with fluorescent labelled antibodies (1 : 500 dilution in blocking buffer). Negative controls were prepared as described above, but no anti-ATP7A or ATP7B was included in the reaction mixture accordingly. Cells were counterstained with 4,6-diamidino-2-phenylindole (DAPI) and imaged with a Leica TCS SP5 Confocal Laser Scanning Microscope (Leica) using a 40× water immersion objective.

### Analysis of steady-state RNA levels

RNA was purified from at least three independent biological replicates of proliferating and differentiated primary myoblasts with TRIzol (Invitrogen). cDNA synthesis was performed with 0.5 μg of RNA as template, random primers, and SuperScript III reverse transcriptase (Invitrogen) following the manufacturer's protocol. Quantitative RT-PCR was performed with Fast SYBR green master mix on the ABI StepOne Plus Sequence Detection System using the primers listed in ESI† Table S1 and normalized to the levels of *Ef1-α* expression. Importantly, Cu treatment does not affect the mRNA levels of *Ef1-α*, *C*_t_ values for this transcript remained constant in all conditions tested. Change in gene expression was calculated using the comparative *C*_t_ method.^98^ Two-tailed t-tests were performed for statistical analyses using Kaleidagraph software.

### RNA stability analysis

Analysis of mRNA stability was performed using actinomycin D as previously described.^62^ Briefly, cells were treated with 5 ng ml^–1^ actinomycin D to halt transcription for 0.25, 1, 2, 4, 6, or 8 hours. After aspiration of medium, cells were washed once with PBS and were scraped in TRIzol for isolation of RNA. Steady-state levels of targets were then analysed as described above. In all cases *Myc*, an unstable transcript, was used as a control for efficacy of actinomycin D. Primers used are listed in ESI† Table S1.

### Analysis of alternative poly(A) signal (PAS) utilization

To determine changes in PAS utilization, length of the 3′ untranslated region (UTR) was determined using qRT-PCR as described.^99^ Briefly, qPCR was performed using primers designed to regions within the coding sequence or distal 3′ UTR (between proximal and distal PAS sites) listed in ESI† Table S1. Results were calculated using the comparative *C*_t_ method^98^ comparing distal 3′UTR products to total PCR product amplified by coding sequence primers.

### Statistical analysis

All statistical analysis was performed using Kaleidagraph (Version 4.1) or Graph Pad Prism 7.0b. When comparing experimental results with two data points, statistical significance was determined using paired or unpaired Student's *t*-test. When comparing multiple data points, statistical significance was determined using one-way analysis of variance (ANOVA). Experiments where *p* < 0.05 were considered to be statistically significant.

## Conflicts of interest

The authors declare no conflict of interest.

## Supplementary Material

Supplementary information
