# Supplementary material for: Dynamic changes in copper homeostasis and post-transcriptional regulation of Atp7a during myogenic differentiation
Source: Metallomics. 2018 Jan 4;10(2):309–22. doi: 10.1039/c7mt00324b (PMC5824686; doi:10.1039/c7mt00324b)
Supplement: Supplementary file 1 [file MT-010-C7MT00324B-s001.pdf]

Electronic Supplementary Material (ESI) for Metallomics.
This journal is © The Royal Society of Chemistry 2018

SUPPLEMENTATY INFORMATION

Dynamic changes in copper homeostasis and post-transcriptional regulation of *Atp7a* during myogenic differentiation

Katherine E. Vest, Amanda L. Paskavitz, Joseph B. Lee, Teresita Padilla-Benavides*

SUPPLEMENTARY FIGURES

Supplementary Figure 1

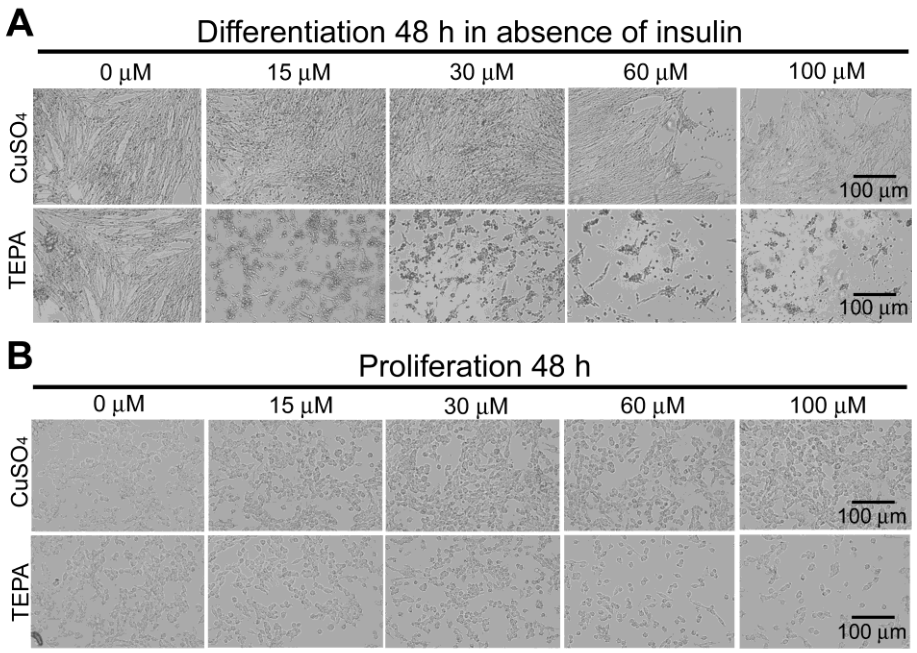

Supp. Fig. 1. Effect of increasing concentrations of Cu in proliferating and differentiating primary myoblasts derived from murine satellite cells. A. Representative light micrographs of myotubes differentiated for 48 h in insulin-depleted media with addition of increasing concentrations of $CuSO_4$ (top) or TEPA (bottom). B. Representative light micrographs of proliferating myoblasts for 48 h cultured with increasing concentrations of $CuSO_4$ (top) or TEPA (bottom).

Supplementary Figure 2

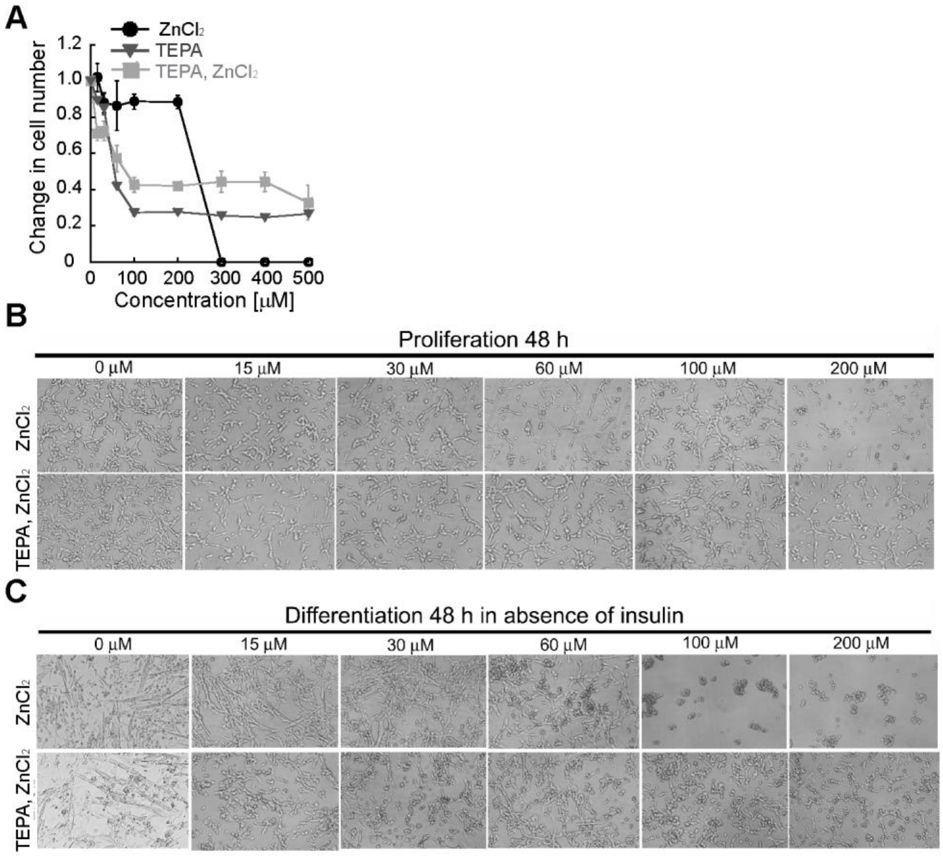

Supp. Fig. 2. Effect of increasing concentrations of Zn in proliferating and differentiating primary myoblasts derived from murine satellite cells. A. Cell counting assay of primary myoblasts grown under the same culture conditions as in panel A. Data represent the average of at least three independent experiments ± SE. B. Representative light micrographs of proliferating myoblasts for 48 h cultured with increasing concentrations of $ZnCl_2$ (top) or TEPA treated cells supplemented with equimolar concentrations of $ZnCl_2$ (bottom). C. Representative light micrographs of myotubes differentiated for 48 h in insulin-depleted media with addition of increasing concentrations of $ZnCl_2$ (top) or TEPA treated cells supplemented with equimolar concentrations of $ZnCl_2$ (bottom).

Supplementary Figure 3

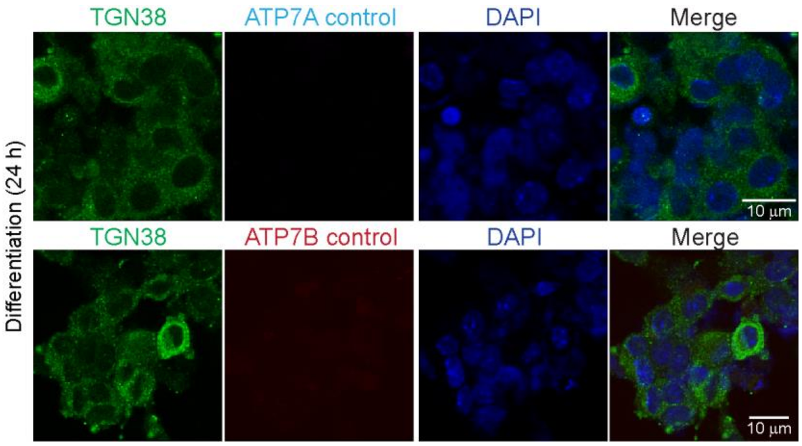

Supp. Fig. 3. Negative controls for immunostaining and confocal microscopy. Representative confocal micrographs of myoblasts differentiated for 24 h immunostained for TGN38. Primary antibodies against ATP7A or ATP7B were excluded from the mixture, and secondary antibodies were later included as a negative control.

Supplementary Figure 4

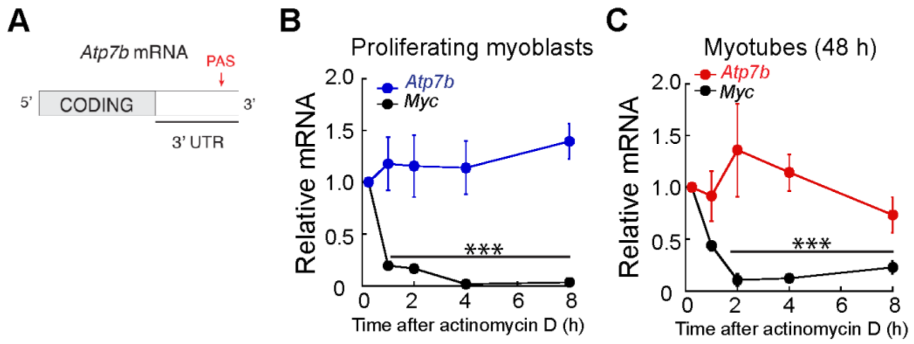

Supp. Fig. 4. Stability of *Atp7b* mRNA does not change during myogenic differentiation. A. Schematic of *Atp7b* 3' UTR showing the presence of only one PAS. B. *Atp7b* mRNA in proliferating myoblasts is stable as determined by actinomycin D treatment followed by qRT-PCR. Stability of *Myc* mRNA was used as a positive control. C. *Atp7b* mRNA in differentiated myotubes is stable as determined by actinomycin D treatment followed by qRT-PCR. Stability of *Myc* mRNA was used as a positive control. All data represent the average of three independent biological experiments ± SE; ****P<0.001*.

Supplementary Table 1. Primers used in this study.

**Primer name**	**Sequence 5’-3’**	**Reference**
EF1α F	AGCTTCTCTGACTACCCTCCACTT	(1)
EF1α R	GACCGTTCTTCCACCACTGATT	(1)
Ctr1 F	GGAATCCGCGGCCTTTACT	This study
Ctr1 R	TCAGTGGAAAAGTCTTCACA	This study
Pax7 F	GCAGCTGGAGGAGCTAGAGAAG	(2)
Pax7 R	GTCTCCTGGCTTGATGGAGTCG	(2)
Myogenin F	CAAGTGTGCACATCTGTTCTAGTCTCT	(1)
Myogenin R	GTATCATCAGCACAGGAGACCTTGGT	(1)
Mhcll F	TCAATGAGATGGAGATCCAGCTGAAC	(1)
Mhcll R	GTCCAGGTGCAGCTGTGTGTCCTTC	(1)
Acta1 F	TTGTGCGCGACATCAAAGAGAAGC	(1)
Acta1 R	GAAACGCTCATTGCCGATGGTGAT	(1)
Ckm F	GCCGGGGATGAGGAGTCCTAC	(1)
Ckm R	GCAGTGCGGAGGCAGAGTGTA	(1)
Atp7a Coding F	CCAAGGGTGTGACTGGTGTT	This study
Atp7a Coding R	GGCACTCACCACAGATGGAA	This study
Atp7a Distal F	TCATCCAGCCCCCTCTATCA	This study
Atp7a Distal R	TCCTGACACAGCACGGATTT	This study
Atp7a Distal 2 F	GCGGGCAACTTTGTGCAATA	This study
Atp7a Distal 2 R	GCGGGCAACTTTGTGCAATA	This study
Atp7b F	AGGAAGAACTTGGCGTCTGT	This study
Atp7b R	CCAACATTGTCGAAGGCGAA	This study

SUPPLEMENTARY REFERENCES

1. Hernandez-Hernandez, J. M., Mallappa, C., Nasipak, B. T., Oesterreich, S., and Imbalzano, A. N. (2013) The Scaffold attachment factor b1 (Safb1) regulates myogenic differentiation by facilitating the transition of myogenic gene chromatin from a repressed to an activated state. *Nucleic Acids Res* **41**, 5704-5716
2. Padilla-Benavides, T., Nasipak, B. T., and Imbalzano, A. N. (2015) Brg1 Controls the Expression of Pax7 to Promote Viability and Proliferation of Mouse Primary Myoblasts. *Journal of cellular physiology* **230**, 2990-2997
